# The Evaluation of a Nursing Care Model for Breast Cancer: What Are Women's Priorities?

**DOI:** 10.1155/jonm/8653274

**Published:** 2025-04-26

**Authors:** Ana Rodriguez-Ortega, Tarsila Ferro, Cristian Ochoa-Arnedo, Gloria Campos, Yolanda Valverde, Joan Carles Medina, Josep Maria Borras

**Affiliations:** ^1^Department of Basic and Clinical Nursing, Faculty of Nursing, Universitat de Barcelona, Barcelona, Spain; ^2^Catalan Institute of Oncology, Barcelona, Spain; ^3^Department of Clinical Psychology and Psychobiology, Faculty of Psychology, Universitat de Barcelona, Barcelona, Spain; ^4^Bellvitge Biomedical Research Institute (IDIBELL), Barcelona, Spain; ^5^Department of Psychology and Education Sciences, Open University of Catalonia, Barcelona, Spain; ^6^Department of Clinical Sciences, Faculty of Medicine and Health Sciences, Universitat de Barcelona, Barcelona, Spain

**Keywords:** breast cancer, breast care nurse, nurse, nurse's model, patient satisfaction

## Abstract

**Aim:** To assess patient satisfaction with the breast care nurse (BCN) model and its adequacy in meeting patients' needs for information and support.

**Background:** The BCN is a core multidisciplinary member of the breast cancer team. The evaluation of care models is necessary to detect gaps and improve the quality of care.

**Material and Methods:** This cross-sectional descriptive study took place in a breast pathology unit and included patients with early breast cancer seen between 1 July 2016 and 30 June 2017 after finishing their treatment. Between July and December 2018, sociodemographic and clinical variables were collected from the clinical history, and satisfaction was measured using a questionnaire sent to the patients.

**Results:** Of the 139 patients included, 99.3% reported that the BCN provided information correctly, 96.2% reported that she provided adequate information on self-care at home (96.2%), and 97.8% reported that the words of the BCN helped them feel better. However, some patients were unsure whether the BCN would have been willing to discuss alternative therapies (41%).

**Conclusions:** Patients were satisfied with the BCN, including her role in meeting information and support needs. However, some issues needed to be sufficiently addressed. Comprehensive, continuous assessment is required to understand patient needs. Training and specific studies on topics that are of interest to patients can help respond to these needs.

**Implications for Nursing Management:** BCN functions are being developed in some countries. BCN results make it easier for healthcare managers to commit to this role and for nurses to develop all their competencies. BCN models must respond to international guidelines but are also determined by organizational resources. The evaluation of these models is essential and must be considered by users. Advanced practice nursing roles, including the BCN, are well established in some countries but developing in others. BCN results make it easier for healthcare managers to commit to this role and for nurses to develop all their skills. BCN models must respond to the elements determined by organizations that work to improve the quality of care for patients with breast cancer. However, they are also determined by organizational resources. The evaluation of these models is essential to correct deficiencies and improve the quality of care. An important part of the evaluation must take into account the user who receives the care, in terms of satisfaction and the form of patient-reported outcome measures (PROMs).

## 1. Introduction

Breast cancer is one of the most common types of cancer globally and has the highest incidence among the female population. In 2018, there were 2,088,849 diagnosed cases of breast cancer worldwide, which represents 11.6% of total cancer diagnoses or 24.2% of cancer cases in women [1]. These numbers are on the rise, with estimates indicating a 21.1% increase in new cases and a 25.2% rise in breast cancer mortality worldwide within just 10 years, from 2020 to 2030 [2]. In the European Union, the probability of the onset of breast cancer before reaching the age of 75 is estimated at 8%, and the average rates in Spain are similar to those observed across EU member states [3]. According to the Cancer Registry of Catalonia and the tumour registries of Girona and Tarragona, an annual diagnosis count of approximately 4200 cases has been documented, with an incidence rate that has remained stable over the past few years [4–6].

## 2. Background

The literature demonstrates that the early management of breast cancer by a multidisciplinary team of breast cancer specialists improves outcomes in terms of patient survival and quality of life [7, 8]. These multidisciplinary teams, comprising professionals specialized in diagnosing, treating and caring for patients with breast cancer, are systematically organized and coordinated within entities commonly recognized as breast centres or breast units [9–11].

The breast care nurse (BCN) is currently a multidisciplinary breast cancer team member [12]. The figure of the BCN appeared in the 1970s in the United Kingdom, and later in Sweden, the Netherlands, the United States and Australia [13, 14]. The profile originated within the framework of multidisciplinary teams, initially to provide emotional support and advice, and later attributed other responsibilities corresponding to expert clinical professionals [14]. In Australia, in the 1990s, studies highlighted the influence of the BCN in improving care coordination [15, 16]. Despite these advances, the role of the BCN still needs to be defined internationally in terms of standardizing functions, competencies, training and nomenclature. This lack of definition makes it hard to obtain reliable clinical data relating to the care provided by BCNs or to its cost-effectiveness [16, 17].

Nevertheless, based on a review of international position documents on the BCN's role [7, 17–20], common core competencies can be established, which are: holistic needs assessment; provision of health education and information; provision of emotional support; assistance in patient decision-making, support to family and caregivers; coordination of care and facilitation of continuity of care; consultations with other professionals when necessary; and the presence during different phases of the disease: diagnosis, treatment and follow-up.

The provision of care by the BCN in accordance with these core competencies improves the patient's experience and that of the other professionals in the multidisciplinary team [21–23]. It also improves the satisfaction of patients' needs, especially when the BCN is present from the start of the care process [22–26]. Finally, it enhances continuity of care, which probably has a positive impact on clinical outcomes [27, 28].

Several of the BCN competences mentioned above respond to patients' needs for information and support. According to the literature, the results obtained by BCNs indicate that patients express a high level of satisfaction with the information provided by BCNs [21, 22, 28, 29]. Cases are described in which a BCN intervenes in one phase of disease treatment, in which patients feel that their information needs have been satisfied, for instance during diagnosis [30], chemotherapy and radiotherapy [31, 32] and survival care [33], or within certain programmes like genetic counselling [34]. However, new ones appear in phases in which the BCN does not intervene [21]. Indeed, in other care models in which patients do not have access to a BCN, they report such information needs. The authors conclude that a BCN plays an essential role for case management [29]. Communication needs transcend the communication between nurse and patient. The literature shows significant improvements in patients' perception of communication between professionals involved in their care if they have access to a BCN [22]. In a patient's words, ‘nurses are helpful in the clinical sense and to receive all the information needed' [26].

The need for support is also a broad concept encompassing care and emotional, social and spiritual support. The need for information and support can change during a patient's illness, and it requires ongoing monitoring [35]. The literature shows lower figures for anxiety and worry about the future and death when the patient has access to a BCN compared to patients who do not have access to this type of nurse [23, 24]. The BCN facilitates acceptance of the diagnosis and the expression and management of emotions [28, 36].

The care models implemented by the BCN must be based on core competencies and, at the same time, respond to patients' changing needs during the stages of their disease trajectory. To achieve this, the BCN must strategically plan a series of interventions adapted to crucial moments, such as diagnosis, treatment and follow-up. In these interventions, the BCN must address patient-related considerations, including information and support needs, tailored to the unique characteristics of individuals and their families, and their preferences and circumstances.

Existing literature provides examples of care models that align with the aforementioned competencies and intervene strategically at critical moments in the course of the disease [22, 37, 38]. Similarly, some published studies evaluate patient perception within the BCN framework [36, 39], but the components of the patients' care model are not sufficiently detailed.

Overall, the BCN scores well in terms of satisfaction with care. When asked about the benefits of having access to the BCN, patients report ‘better experience' [22], ‘better care' [23] and ‘respect for their preferences' [21]. The BCN makes them feel safe in their disease process and in seeking information. It also makes them feel understood and involved in decisions about their illness [23]. The BCN improves their perception of the healthcare system by facilitating coordination between levels of care [23, 28]. In some studies, 98% of patients would recommend the service provided by the BCN to others [40].

Although the publications do not detail all the elements of the respective care models, they cover most of the core competencies defined for the BCN and patients consider that the models are satisfactory. This study's research team previously carried out a self-assessment study of the BCN care model in a tertiary hospital in Barcelona, in terms of compliance with internationally accepted core competencies of this role, and the results were positive [41].

**Figure 1:**
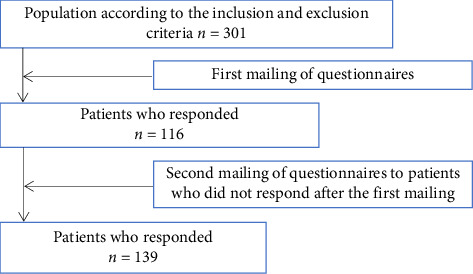
Flowchart with participation data showing the number of patients who took part in the study.

The model of care studied is based on the competencies described for the role of the BCN and is provided to the patient through several visits during the primary treatment (except for radiotherapy, where nursing follow-up is carried out within the radiotherapy oncology service). The BCN visits patients before the start of each of the treatments, to provide health education designed to prevent or reduce the degree of toxicity; performs surgical wound care; during chemotherapy, periodically visits the patient to detect and monitor adverse effects; performs an initial control of tolerance to hormone therapy (2 months after start); and visits the patient once all treatment has been completed (not including hormonal treatment, which has a minimum duration of 5 years). On average, each patient has 9.5 visits with their BCN. During these visits, the BCN assesses the patient's needs and plans the necessary care; provides information about the disease and its treatments; informs about the care circuits and support resources available at the centre; provides health education regarding possible adverse effects; provides emotional support before, during and after the treatments are performed; manages contingencies that arise throughout the disease process through nursing care (drainage of seromas, oncogeriatric screening, counselling, etc.) or by referring the patient to other professionals (the rehabilitation service, psycho-oncology, social work, etc.). At the end of the treatment, a single visit scheduled by the BCN is made, and care is offered on demand, for the following years with no time limit [41]. Each patient is assigned to one of the three BCNs of breast functional unit (BFU), who is responsible for them throughout the disease. During this time, the BCN will perform direct patient care activities and manage their case, including diagnostic tests and visits from the different professionals of the multidisciplinary team, presenting the case to the psychosocial committee in case of psychological and/or social complexity, or participating in the tumour committee. The standardized BCN contacts, according to the treatments received by the patient and their sequence, and the competencies performed by the BCN in each of the encounters are shown in Figure 1 and Table 1 of the additional files. Additional Figure 1 & Table 1.

The objective of this study is to ascertain whether this model is also capable of fulfiling patients' informational and support requirements and expectations.

## 3. Aim

1. To evaluate the general satisfaction of patients with the BCN model under study.
2. To evaluate whether the BCN model that was studied meets patients' information and support needs.

## 4. Methods

### 4.1. Study Design and Participants

This cross-sectional descriptive study was conducted at the BFU of the Catalan Institute of Oncology, a specialized cancer centre, and University Bellvitge Hospital, a tertiary hospital in the metropolitan area of Barcelona.

The study's inclusion criteria involved all patients from the BFU who had completed their primary treatment. Patients with a first visit to the BFU between 1 July 2016 and 30 June 2017, who were diagnosed with early breast cancer, either for the first time or after a local relapse, were included. The exclusion criteria were diagnosis of benign phyllodes tumour, lobular carcinoma in situ, sarcoma and advanced breast cancer; receiving part of the treatment at another centre; and the patient's decision not to undergo surgical treatment.

### 4.2. Measures and Instruments

Two questionnaires were used to measure the study variables. The first questionnaire was prepared ad hoc for the present study and contained sociodemographic and clinical data, including age, gender, employment status, living situation, economic condition and social support, clinical stage of the disease, initial diagnosis or recurrence and treatments received.

The second questionnaire was the Ipswich Patient Questionnaire. This questionnaire was developed to assess the support provided by BCNs to patients with early breast cancer in rural and remote areas of Australia [39]. The questionnaire is not a psychometric tool, so validation was performed only at face and content levels to ensure that the questions were understandable and unambiguous. The questionnaire was later validated in a multicentre study in Israel to understand the perceptions of BCNs among women with breast cancer [36]. Before the start of this study, permission was obtained from the research team that developed the questionnaire for its use.

The questionnaire refers to specific details of the BCN care model, such as the patient's first contact with the BCN and ease of access to this service. The questions that evaluate the quantity and quality of the information and support provided by the BCN have Likert-type responses, such as strongly agree, agree, disagree, strongly disagree and not sure; or answers such as did not inform me, I wanted more, right amount, I wanted less and not sure. The questionnaire also refers to the connection with other professionals and the patient's assessment of the personal characteristics of the BCN. The questionnaire is administered once treatment is completed.

For the present study, the Ipswich Patient Questionnaire was slightly modified by adding and eliminating some questions to adapt it to the Spanish healthcare model and the BCN model. Two questions were added: the first asking whether the patient would want to be treated again by the same team, if necessary; and the second allowing additional comments to be added. Questions related to housing issues (rural and remote) and insurance companies were excluded, as the patients seen are drawn from a relatively limited geographical area and the centre is publicly funded. It was translated into the Spanish and Catalan languages.

Before use, the questionnaire was tested by an expert patient, who collaborated on different projects at the research centre, to check the intelligibility of the questionnaire and aspects related to ease of answering or the lack of ambiguity of the questions.

### 4.3. Data Collection

The Ipswich Patient Questionnaire, an information sheet and informed consent were sent to the study population by postal mail. After 2 weeks, a second postal mail was sent to patients who had not responded. The Ipswich Patient Questionnaire was sent between July and December 2018. When the signed informed consents and questionnaires completed by the patients were received, data relating to their sociodemographic characteristics and clinical profile were extracted from their medical records.

### 4.4. Statistical Analysis

Data were introduced and curated in a database and then analysed using the SPSS version 21 (IBM) statistical package.

First, participants who completed the Ipswich Patient Questionnaire and those who did not were compared with regard to their sociodemographic and clinical characteristics using the chi-square test for categorical variables, such as education level or lack of social support, and the Mann–Whitney *U* test for continuous variables, such as age or time elapsed from diagnosis to the assessment. Then, responders' characteristics were reported using descriptive statistics, categorical variables are expressed as frequencies and percentages, while continuous variables are reported as median and range, according to the non-normality of the data.

Finally, Spearman's correlation coefficient was used to assess the association between age and questionnaire responses (i.e., patient satisfaction with the information and support received from the BCN); for the remaining sociodemographic and clinical variables, which were categorial, the Mann–Whitney *U* and the Kruskal–Wallis tests were used as appropriate.

Statistical tests with a *p* value < 0.05 were considered statistically significant.

## 5. Results

### 5.1. Results on Participation and Sociodemographic and Clinical Data

Four hundred and twenty-five patients were originally enroled in the study. After application of the exclusion criteria, the study population comprised 301 patients, of whom 139 (46.18%) responded to the questionnaire.

Figure 1 details the patients according to the inclusion, exclusion and acceptance criteria for participation in the study.

An analysis of the groups of patients who responded to the questionnaire and those who did not respond revealed a difference in the time elapsed since the end of treatment. Patients who filled in the questionnaire had completed treatment more recently (*U* = 11,155, *p* = 0.039). Among responders, the average time elapsed from the diagnosis of breast cancer to the completion of the questionnaire was 2.37 years (SD = 0.42).

The sociodemographic and clinical data are shown in Table 1.

### 5.2. Results of Patient Satisfaction With the BCN Model

In response to objective 1 of the study, to evaluate the general satisfaction of patients with the BCN model studied, the following results were obtained.

A total of 52.6% of the patients stated that their first contact with the BCN was at the time of diagnosis or a couple of days later, and 30.7% reported that it was before the first treatment. A total of 96.4% of the patients believed that the timing of the first contact with the BCN was appropriate. The model includes the first visit to the BCN after the diagnosis and the treatment decision in the committee, which delays this visit by around 2 weeks compared to the first visit to the BFU.

Of the patients, 43.9% were visited by a single BCN in the breast cancer care unit, and 33.1% were visited by more than one, but usually by their BCN. A total of 93.6% believed it was better if the same BCN visited them every time. At the BFU, there are three BCNs and each one is usually responsible for visiting their patients. However, for organizational reasons, in the unit, the BCN who cares for the patient during the pre and postsurgical process may not be the one who cares for the patient during the chemotherapy process, for example.

Of the patients, 81.9% believed contacting the BCN was easy when needed. A total of 99.2% felt more secure/calm because they could contact their BCN by phone or email. Patients have the direct mobile phone number of their BCN or their email.

### 5.3. Results on the Satisfaction of Information and Support Needs

In response to objective 2 of the study, it was assessed whether the studied BCN model meets patients' information and support needs. The patient's responses can be seen in Tables 2 and 3.

The BCN visits the patient before each treatment to assess her needs, provide health information and education and offer support according to her needs. The BCN also carries out proactive monitoring with in-person or telephone appointments to assess toxicities related to the treatments or the evolution of detected needs.

The patients believed that the BCN provided the correct amount of information on how to take care of themselves at home (96.2%) and practical information (92.8%). However, there needed to be more information provided on treatment options (12.4%) or external support services (16.9%). At the BFU, information about the recommended treatment is provided by the doctor. The BCN helps patients to understand this information, but usually, it has already been received when the patient is visited by the BCN. The nurse requests a new visit with the doctor when the patient has doubts about the decision that has been made or if she has changed her mind.

The patients agreed or strongly agreed that the BCN provided the right information (99.3%). However, 37.4% of the patients were unsure about whether the BCN had provided information on where to find spiritual support. The hospital where this study was conducted lacks specific resources to provide spiritual and religious care to patients, nor are these needs directed to other external resources.

In addition, patients agreed or strongly agreed that the BCN told them things that helped them feel better (97.8%).

The association of sociodemographic and clinical variables with questionnaire responses (both satisfaction with the BCN model and with information and support received) were analysed but revealed no statistically significant results.

### 5.4. Results Related to Other Aspects of the Role of the BCN

A total of 41% of patients were unsure whether the BCN would be willing to discuss alternative therapies, and 24.5% were uncertain whether the BCN had helped them communicate their needs to other professionals. In the written and protocolized information provided to patients, no specific guidelines are given on complementary therapies. The hospital organizes workshops for patients on mindfulness or meditation, for example, but there are no specific workshops to address the topic of complementary therapies and their compatibility with oncological treatments.

According to patients, the BCN advised 10.8% of patients to schedule visits with social workers, 24.5% with psycho-oncology, 28.1% with specialist doctors, 34.5% with infusion area or radiation oncology nurses, 22.3% with their primary care doctor or nurse and 22.3% with patient groups or associations. BCNs of the BFU make independent decisions on referrals to other professionals. In addition , they participate in the weekly psychosocial committee to discuss cases with complex psychological, social or economic needs.

According to the patients, the most important personal characteristics that the BCN should possess are being a good professional and conveying security (75.5%); being good and practical at decision-making (64.7%); and being sensitive, compassionate, empathetic and understanding (63.3%). The least essential personal characteristics of the BCN, according to patients, are having authority within the team (9.4%) and respecting privacy (5%). A total of 99.3% of patients would like to be treated in the centre itself or by the same professionals, if necessary.

Finally, the last question offered the opportunity to add a comment. A total of 72% of patients answered, with most comments expressing gratitude or satisfaction. Some comments were directed at their BCN:

‘My relationship with my nurse was very close, direct, and attentive; professional and sensitive'.

‘My nurse is very competent, empathetic and a true professional. She has been of great help'.

‘Thank my nurse for her support and for being there when I needed her'.

‘My gratitude to my nurse… who had the patience to… explain and reassure me…'

## 6. Discussion

The EUSOMA working group proposes that the first visit with a BCN should be carried out at the time of diagnosis or within a maximum period of 1 week [19]. Furthermore, according to the literature, if this visit is carried out at the beginning of the care process, we can see improvements in the satisfaction of the needs [22].

The study results show patient satisfaction with the type and timing of access to the BCN. In response to the question of when the initial visit to the BCN was made, over half of the patients indicated that it occurred at the time of diagnosis or within a couple of days thereafter. However, this is inaccurate. In the model under examination, patients are typically visited by the BCN for the first time approximately 3 weeks after diagnosis [41]. The error in the patients' response is probably because of the time elapsed between diagnosis and response to the questionnaire (over 2 years). Despite this, 96% of patients considered that the timing of the first visit with the BCN was appropriate. Patients are satisfied with the timing of this initial visit, but the model has been modified to comply with EUSOMA's recommendation. Thus, the first visit from the BCN is scheduled for the first week.

Patients establish a relationship of trust with the BCN. This trust is reflected in both the comments provided and the responses given regarding the personal characteristics that patients value most about the BCN. These characteristics were identified as professionalism and closeness. The preference of patients is for a single BCN to oversee their entire care process; however, this is not always a possibility. In some cases, one BCN conducts both the pre- and postoperative follow-up of the patient, while another BCN from the same team provides follow-up during the patient's chemotherapy treatment. Furthermore, during holiday periods, BCNs collaborate to ensure comprehensive care coverage, necessitating adjustments in schedules. This sometimes results in difficulties for the same BCN in maintaining patient follow-up.

Ahern et al. [24] studied unmet needs in patients with access to a BCN. The most frequently mentioned needs to which patients gave the most importance were fear of relapse, having access to a professional on the team if they needed it, symptoms of anxiety or depression, lack of energy and changes in their sexual behaviour. In the present study, the majority of patients expressed satisfaction with the quantity and quality of information provided by the BCN, which is consistent with the results of studies using the same questionnaire [36, 39] and with the literature that evaluates BCN results [21, 22, 28, 29, 42]. Regarding satisfaction with the need for support in women with breast cancer, various studies demonstrate that the BCN achieves positive results [21, 23, 29, 36, 40, 42]. This study shows that the BCN helps the patient to feel better and cope with the diagnosis and facilitates the expression of feelings and communication between the patient and other team members. In addition, the BCN provides this support when it is most needed.

However, according to patients' responses, certain informative needs were not met. In particular, there was a lack of information about external support services, and they had doubts about whether the BCN provided them with information about where to obtain spiritual help, about the use of alternative therapies or about treatment options.

Regarding the result obtained on the lack of information on external support services, Kadmon et al. [36] found a similar result. The literature shows that healthcare for cancer patients often suffers from fragmentation and lack of coordination [43, 44]. This fragmentation also carries over to support resources. The Catalan Institute of Oncology, as a monographic oncology centre, has numerous resources to support patients. However, some people may prefer to learn about other resources. The BCN must be aware of the available community resources and, in the process, improve the management of health resources.

As has been previously observed, many patients were unsure whether the BCN had informed them where to find spiritual support. In the study by Kadmon et al. [36], almost half of the patients indicated that they did not receive sufficient information regarding spiritual support. However, the study concluded that the patients' levels of religious belief did not affect their perception of the BCN. The literature reveals that individuals who have undergone treatment for breast cancer frequently raise spiritual and religious questions, including those pertaining to the meaning of life [45]. To ensure comprehensive care, it is essential for the BCN to assess these concerns and each patient's coping strategies and to provide support in the search for answers and information about specific resources.

As previously indicated, a notable proportion of patients were uncertain as to whether the BCN was amenable to engaging in conversations pertaining to alternative therapies. This result is also consistent with that found by Kadmon et al. [36]. Cancer patients are increasingly using complementary therapies to minimize the adverse effects of treatments [46]. However, some of these therapies are incompatible with oncological treatments. It is necessary to educate patients about their use. Doctors and nurses working in oncology need to know the scientific evidence in this field to offer appropriate recommendations to patients.

Another outcome that was highlighted was that a few patients indicated that they had not been made aware of the various treatment options by the BCN. Although guidelines recommend shared decision-making [47, 48] in many other countries, it is not carried out very frequently [49, 50]. However, an increasing number of people are seeking information about cancer and its treatments [51]. Therefore, the BCN should ask patients about this issue and offer a space to discuss the available options or explain the reason for their treatment recommendation. A qualitative methodology study might have allowed us to provide a more nuanced view of unmet information and support needs or what interventions on the part of the BCN would have been appreciated. A methodology of this kind could offer relevant information on areas with room for improvement in the care model.

On the other hand, the results of the study show how BCNs consult other professionals. Continuous monitoring allows the BCN to know the needs of the patients at the moment when these needs arise, and to be able to intervene with nursing care or refer to the appropriate professionals, when the situation requires it. In this way, the BCN becomes a connection point between the patients and the different specialists. The literature shows that continuous monitoring and referral provides patients with a sense of security and that teams are well coordinated and efficient [22, 28, 29, 36, 40].

As previously discussed, the role of the BCN first emerged in the 1970s. In some countries, the BCN's role is clearly defined and regulated, with standards set for training and experience required. In the 2000s, Australia and the United Kingdom established a framework for defining the competencies required of the BCN. The standards set out the competencies and expected levels of knowledge, skills, attitudes and behaviours for the BCN's role [35, 52]. Nevertheless, many countries do not formally recognise the BCN or regulate it. The present study was conducted in Spain where the BCN is not recognised. In 1995, the ICO incorporated the BCN. The number of nurses has been augmented to three to respond to an increase in BFU patient numbers and the range of competencies they assume. The BCN's role is based on the advanced practice nursing model, which allows for the development of the nursing profession [17, 20, 53, 54]. Almost 3 decades after the BCN was integrated into the BFU, its care model was evaluated. This was done from two perspectives. Initially, the BFU's BCN model was examined for compliance with BCN competencies [7, 17–20, 28].

The model has been updated following the study findings. The nurse visit has been brought forward to the first week after diagnosis, enabling support before treatment is decided. This helps with emotional distress and lack of info at this vulnerable time. Secondly, before starting treatment, patients are asked about their concerns regarding the proposed treatment. They are also told about the benefits and risks of alternative therapies. Furthermore, information on external support resources, including community resources and reliable reference websites, is being collected and made available to patients. Because of limited resources, post-treatment care by the BCN is limited to a single scheduled visit 2–3 months after treatment (excluding hormone treatment, recommended for at least 5 years) and unscheduled visits requested by patients. To fill the gap in post-treatment support, a monthly group workshop, led by a BCN, has been set up for patients who have finished their treatment. It covers healthy lifestyle habits, emotional support, sexuality, cancer control and participation in screening programmes.

In order to ascertain patients' opinions of the fundamental keys of the model and extrapolate these findings to other centres, a study employing qualitative methodology is proposed. This study would facilitate the investigation of a range of topics, including spiritual aspects.

This study focuses on patients with early stage breast cancer. We also need to find the most effective model of care for patients with advanced breast cancer, but there are only few tools suitable for this demographic. To do this, a mixed-method study is proposed, which would include a qualitative component in which patients discuss which elements of the BCN meet their needs, followed by a questionnaire with this information. This would be followed by a quantitative study to assess whether the current BCN model meets the needs of these patients.

The extant literature illustrates that the BCN is efficacious in a variety of domains, addressing both physical and emotional needs and enhancing satisfaction with the health system, among other outcomes. However, there is little evidence on how adding the BCN to multidisciplinary teams affects survival rates [27, 28]. One potential explanation for this is that the BCN operates within a multidisciplinary team, which makes it challenging to discern the impact of its intervention on the outcomes achieved by the entire team. It seems reasonable to posit that the inclusion of a BCN in a multidisciplinary team contributes to the benefits achieved by the team as a whole, with results that are already well established.

One of the core functions of the BCN is to encourage the adoption of healthy lifestyle habits among its patient population. It is well established that these lifestyle habits help to reduce the risk of treatment-related complications. Furthermore, they also reduce the risk of disease relapse and the appearance of other cancers and chronic diseases [55–57]. An indirect approach to measuring the impact of the BCN on survival rates could involve examining whether the effective incorporation of healthy lifestyle habits increases survival in patients who are cared for by a BCN compared to those who do not have this professional in their breast cancer teams. This could yield insights into the health outcomes associated with these BCNs.

### 6.1. Limitations

This study has some limitations. First, it does not compare a group attended by a BCN with a control group. Although this design would have increased the power of the results, it was discarded. This is because the literature supports the benefits of access to a BCN, and the model that was studied always provides access to a BCN. Therefore, it was considered unethical to withhold access. To improve the power of the results, the study could be reproduced by comparing the UFM with another breast pathology unit that does not yet have BCN.

Secondly, the model that was studied is used in a single centre. The Catalan Institute of Oncology of Hospitalet is a dedicated cancer care centre in the public health system of the southern metropolitan area of the city of Barcelona. It has all the services necessary for comprehensive cancer care. The study population are of low socioeconomic status and are unlikely to have private insurance, and they also prefer public healthcare for serious health problems, such as cancer. Most of the cancer care in Spain is carried out in the public sphere, largely because of the high degree of trust that the public system generates in the population. A priori the results cannot be extrapolated. However, the Catalan Institute of Oncology has two other breast units in its centres located in other towns and works in a network with 19 regional hospitals, which means it is the reference centre in oncology for 45% of the adult population of Catalonia, that is, more than 2.8 million people. Once areas for improvement have been identified and modifications made, the model will be replicated in the rest of the breast units of the Catalan Institute of Oncology and the centres of its healthcare network, which will multiply its current impact. To extrapolate the model, it would be useful to conduct in-depth interviews with patients, to ask them about the elements of the model that most influence their satisfaction and the meeting of their needs.

Another limitation is that the Ipswich Patient Questionnaire is not a psychometric tool. In addition, it has been translated and slightly modified to adapt to some elements of the model evaluated. It is pending validation for our language and the aforementioned adaptation. Since the questionnaire used has not been validated, it only allows a comparison of the results obtained with those of the two other studies referenced but not with research that had used a different and validated instrument [36, 39]. Despite this, it was chosen because it offered information on patient satisfaction with very specific aspects of the BCN's role, which other validated questionnaires could not offer. Furthermore, no information is available on the patients who did not participate. It has been observed that patients who completed their treatment long ago responded less than those who had been treated more recently. However, it cannot be ruled out that there are other reasons, such as that they were not satisfied with the care provided by the BCN.

Finally, this study only considers patients with early stage disease, since the Ipswich Patient Questionnaire refers to patients who have completed treatment, so it is not applied to patients with advanced disease. We do not have information on the satisfaction of patients with advanced disease with the BCN care model. To study satisfaction with BCN among patients with advanced-stage breast cancer, the questionnaire will have to be modified. Between both populations, there are differences when we consider symptoms related to the disease or psychosocial needs, for example, which vary from those of patients with early disease. Perhaps, it would be appropriate to separate patients with very advanced disease or stable disease into subgroups: with acute toxicity or without relevant toxicity or with psychosocial differences such as the need for a permanent caregiver or not.

## 7. Conclusions

Patients who are cared for according to the BCN model studied expressed satisfaction with the care received. They recognise the BCN as a competent, accessible professional. In addition, the BCN's role meets patients' need for information and support. It facilitates understanding of the healthcare environment, disease and treatments. It provides support in various aspects and at different moments of the illness.

However, the BCN needs to sufficiently address some issues, such as complementary treatments or aid in decision-making. The BCN must address patients on the issue of the use of these therapies, their indication and interactions and must provide resources with safe information. Decision-making must be a shared process. Patients should have the opportunity to comment on their own treatment. To do this, the BCN and the rest of the multidisciplinary team have to provide sufficient information adapted to the capabilities of each patient, offer the available options and facilitate understanding of the reason for the recommendations made.

An integral, continuous assessment of the patient's needs facilitates identification of the need for information and support. BCN training and specific studies on topics of interest to patients can provide answers to these needs. Continuing training in counselling techniques or training focussed on emerging topics, such as evidence for complementary therapies in oncology, could improve communication with patients. Carrying out studies with qualitative methodology, which delve into specific aspects of information and support needs, could provide information on how to improve aspects of the model that do not adequately satisfy some of these needs.

However, studies should be carried out to assess the satisfaction of patients' needs and overall satisfaction in the case of advanced disease using specific questionnaires for this type of population.

There is clear evidence about the benefits of BCN care. However, there are aspects that have not been sufficiently explored. Defining a care model and evaluating it each point by point can help establish a solid model that responds to the patient's needs and satisfaction, the efficient coordination of the multidisciplinary team and the sustainability of the health system.

## Figures and Tables

**Table 1:** Sociodemographic and clinical characteristics of the study sample (*n* = 139).

Variable	Patients	
Age in years, median (range) at the moment of diagnosis		
54		(31–88)

Age in years, median (range) at the moment of completing the questionnaire		
57		(33–90)

Sex	*N*	%
Female	137	98.6

Education level		
University studies	26	18.7
Secondary education	55	39.6
Elementary education	45	32.4
Without studies	12	8.6
Missing data	1	0.7

Living situation		
Lives alone	20	14.4
Lives with husband/partner	45	32.4
Lives with children	13	9.4
Lives with husband/partner and children	57	41
Lives with parents	1	0.7
Others	2	1.4
Missing data	1	0.7

Financial concerns/worries	4	2.9

Lack of social support	29	7.6

Stage		
0	30	21.6
IA	34	24.5
IB	2	1.4
IIA	38	27.3
IIB	10	7.2
IIIA	9	6.5
IIIB	12	8.6
IIIC	4	2.9

Recurrence	19	13.7

Surgery		
Yes	139	100

Immediate reconstruction/oncoplasty	79	56.8

Armpit surgery	123	88.5
Armpit surgery type		
Sentinel lymph node biopsy	83	59.7
Axillary lymph node dissection	40	40.3

Chemotherapy	64	46
Type of chemotherapy		
Neoadjuvant	39	60.9
Adjuvant	25	39.1

Radiotherapy	120	86.3
Radiotherapy type		
Adjuvant	106	87.6
Intraoperative	8	6.6
Intraoperative and adjuvant	6	5.8

Endocrine therapy	101	72.7
Type of endocrine therapy		
Neoadjuvant	6	5.9
Adjuvant	96	94.1

Participation in clinical trial	35	25.2

**Table 2:** How patients rated the amount of help and information offered by the BCN (*n* = 139).

	Not inform me		Wanted more		Right amount		Wanted less		Not sure	
	%	*n*	%	*n*	%	*n*	%	*n*	%	*n*
Help provided by the BCN to…										
…understand the roles of the different people involved in my treatment			6.5	9	82.7	115	0	0	10.8	15
…understand the information I received from physicians and health workers			5.8	8	87.8	122	0.7	1	5.8	8
…communicate my needs to other health workers			4.3	6	70.5	98	0.7	1	24.5	34
…make appointments for me			3.6	5	74.1	103	0.7	1	21.6	30
…make transition to the next treatment stage easy			6.5	9	75.5	105	0.7	1	17.3	24

Information offered about…										
Breast cancer	6.7	9	4.4	6	88.9	120	0	0	0	0
Treatment choices	12.4	16	3.1	4	84.5	109	0	0	0	0
The treatment itself	7	9	1.6	2	90.6	116	0.8	1	0	0
Adverse effects of the treatment	6.9	9	4.6	6	86.2	112	2.3	3	0	0
Caring for myself at home	1.5	2	2.3	3	96.2	125	0	0	0	0
Support services	16.9	20	1.7	2	81.4	96	0	0	0	0

**Table 3:** How patients rated the quality of the information and the psychological support provided by the BCN (*n* = 139).

	Strongly agree		Agree		Disagree		Strongly disagree		Not sure	
	%	*n*	%	*n*	%	*n*	%	*n*	%	*n*
The BCN…										
…provided information that allowed me to share my feelings	27.3	38	57.6	80	0.7	1	2.9	4	11.5	16
…was good at explaining things	41.8	56	57.5	77	0.7	1	0	0	0	0
…gave me too much information	7.2	10	29.5	41	39.6	55	8.6	12	15.1	21
…said things that helped me cope or feel a little better about things	40.5	53	57.3	75	2.3	3	0	0	0	0
…offered information and support at the times when most needed	30.9	43	51.1	71	7.2	10	0	0	10.8	15
…offered sufficient information of where to seek spiritual support	11.5	16	30.9	43	17.3	24	2.9	4	37.4	52
…was open to discuss alternative therapies with me	7.9	11	34.5	48	10.8	15	5.8	8	41	57

Support by the BCN helped me…										
…to deal with my diagnosis	32.4	45	51.1	71	10.1	14	0.7	1	5.8	8
…to make treatment choices	15.1	21	51.8	72	15.8	22	1.4	2	15.8	22
…with communication with my physician	28.1	39	53.2	74	8.6	12	0	0	10.1	14
…to deal with concerns my family had	18.7	26	50.4	70	13.7	19	2.2	3	15.1	21
…to express and manage my feelings	20.1	28	51.8	72	12.2	17	3.6	5	12.2	17
…to deal with adverse effects	28.1	39	52.5	73	7.9	11	0.7	1	10.8	15

## Data Availability

The quantitative data used to support the findings of this study are available from the corresponding author upon request.

## Supporting Information

The standardized BCN contacts, according to the treatments received by the patient and their sequence, and the competencies performed by the BCN in each of the encounters are shown in Figure 1 and Table 1 of the additional files.
