# Supplementary material for: The Evaluation of a Nursing Care Model for Breast Cancer: What Are Women's Priorities?
Source: J Nurs Manag. 2025 Apr 26;2025:8653274. doi: 10.1155/jonm/8653274 (PMC12049246; doi:10.1155/jonm/8653274)
Supplement: Supporting Information — Additional supporting information can be found online in the Supporting Information section. [file 8653274.f1.docx]

The BCN model is shown in Figure 1 of the additional files and Table 1 of the additional files.

**1st visit to BFU**

Diagnosis and Staging

**Multidisciplinary team (MDM) meeting**

Treatment decision

**1st treatment surgery**

1^st^ pre-treatment contact by BCN

Subsequent contacts after surgery

Visit by BCN, surgeon and oncologist

**1st treatment neoadjuvant chemo**

1^st^ pre-treatment contact by BCN

Subsequent phone contact 2-3 days after

initiation of treatment

On change in chemo regimen

**1st treatment neoadjuvant hormone therapy**

1^st^ pre-treatment contact by BCN

**MDM meeting**

Decision on adjuvant treatment

**MDM meeting**

Decision on treatment

**Adjuvant chemo**

BCN visit prior to treatment

Subsequent phone contact 2-3 days after initiation of treatment

On change in chemo regimen

**Adjuvant hormone therapy**

BCN counselling at 1 month after initiation

**Adjuvant radiation therapy**

Visits by radiation oncology nursing team

**Surgery**

BCN pre-treatment visit

Subsequent contacts after surgery

Visit by BCN, surgeon and oncologist

**Adjuvant hormone therapy**

BCN counselling at 1 month after initiation

**Adjuvant radiation therapy**

Visits by radiation oncology nursing team

**End of treatment**

BCN follow-up at 2-3 months after completing treatment

**Adjuvant hormone therapy**

BCN counselling at 1 month after initiation

**MDM meeting**

Decision on adjuvant treatment

**Adjuvant radiation therapy**

Visits by radiation oncology nursing team

**Figure 1 of the additional files.** **Breast Care Nurse (BCN) model in the Functional Breast Unit (BFU) of the Institut Català d'Oncologia: BCN visits according to the treatment administered to patients.** (Adapted from Rodriguez-Ortega et al., 2023)

**Table 1 of the additional files. Breast Care Nurse model at the Functional Breast Unit of the Institut Català d'Oncologia: BCN visits according to the treatment administered to patients.** (Adapted from Rodriguez-Ortega et al., 2023).

| **BCN competencies** | **After diagnosis** | **During treatment** | **Post-surgery** | **End of treatment** |
| --- | --- | --- | --- | --- |
| Comprehensive needs assessment |  |  |  |  |
| Emotional support to patient and caretaker |  |  |  |  |
| Health education, on: |  |  |  |  |
| Breast cancer |  |  |  |  |
| Treatment |  |  |  |  |
| In case of surgery: surgical wound care |  |  |  |  |
| Self-care and warning signs |  |  |  |  |
| Healthy behaviors |  |  |  |  |
| Information |  |  |  |  |
| Circuits |  |  |  |  |
| Resources in and outside of hospital |  |  |  |  |
| Pathological anatomy results |  |  |  |  |
| Help with decision-making |  |  |  |  |
| Collaboration with other professionals, normally: |  |  |  |  |
| Psycho-oncology, social work |  |  |  |  |
| Rehabilitation, physiotherapy |  |  |  |  |
| Surgery |  |  |  |  |
| In case of surgery: |  |  |  |  |
| Wound dressing |  |  |  |  |
| Surgical preparation if needed |  |  |  |  |
| Post-surgical follow-up |  |  |  |  |
| Preoperative check |  |  |  |  |
| Schedule follow-up wound care until healing |  |  |  |  |
| In case of chemotherapy: |  |  |  |  |
| Oncogeriatric screening, if needed |  |  |  |  |
| Schedule follow-up to monitor toxicity |  |  |  |  |
| Referral to day hospital nursing services |  |  |  |  |
| Management of central lines |  |  |  |  |
| Monitoring of toxicity |  |  |  |  |
| Schedule follow-up to monitor toxicity |  |  |  |  |
| In case of hormone therapy: |  |  |  |  |
| Monitoring of toxicity |  |  |  |  |
| Promote treatment adherence |  |  |  |  |
| Schedule end-of-treatment visit |  |  |  |  |
